# Supplementary material for: Maize Herbivore-Induced Volatiles Enhance Xenobiotic Detoxification in Larvae of Spodoptera frugiperda and S. litura
Source: Plants (Basel). 2024 Dec 27;14(1):57. doi: 10.3390/plants14010057 (PMC11723000; doi:10.3390/plants14010057)
Supplement: Supplementary file 1 [file plants-14-00057-s001.zip › plants-3354189-supplementary.pdf]

Supplemental Materials

Table S1. Specific primer for real-time quantification PCR

Table S2-9. Details about statistics analyses including F-values, df, and P-values are provided.

Figure S1. Schematic diagram showing the device of larval exposure of *Spodoptera frugiperda* and *S. litura* to herbivore-induced plant volatiles (HIPVs) from maize plants.

Figure S2. Schematic diagram showing the device of larval exposure of *Spodoptera frugiperda* and *S. litura* to volatile cis-3-hexen-1-ol.

Table S1. Specific primer for real-time quantification PCR

Gene	Specific primer (5’- 3’)
GAPDH-F	ATGCTCCCATGTTCGTCGTT
GAPDH-R	CAGAGGGTCCGTCAACAGTC
SfUGT33F28-F	GTGGGCAGAGTACCTCGAAC
SfUGT33F28-R	AGTCACCGTACTTCCAAATACCA
SfUGT40L8-F	AGCAAACATCGCTGGTTACC
SfUGT40L8-R	TAAATGACGCCGTGTTTCGC
CYP4d8-F	ATGAGACTGTACTGCGCTGT
CYP4d8-R	CAGTTCCGCTTGCCAATACC
CYP6B6-F	CTTACTTGGAGTGCGCCTTC
CYP6B6-R	CTTGGTTCTGTCGTCCACCT
CYP4V2-F	AGCAAGGTCATGTCGGATCA
CYP4V2-R	GTGTCATTGTACAGCCACGG

Table S2. Independent sample T-test result in Figure 1

Figure	Pest	Group	d.f.	*F* value	*P* value
Figure 1A	*S. litura*	Control-vs-HIPVs-exposed	48	0.02	**< 0.01****
	S. frugiperda	Control-vs-HIPVs-exposed	48	0.16	0.84
Figure 1B	*S. litura*	Control-vs-HIPVs-exposed	48	58.22	**0.03***
	S. frugiperda	Control-vs-HIPVs-exposed	48	0.49	0.88
Figure 1C	*S. litura*	Control-vs-HIPVs-exposed	48	14.79	0.54
	S. frugiperda	Control-vs-HIPVs-exposed	48	0.03	**< 0.01****
Figure 1D	*S. litura*	Control-vs-HIPVs-exposed	48	8.81	**< 0.01****
	S. frugiperda	Control-vs-HIPVs-exposed	48	0.05	**< 0.01****
Figure 1E	*S. litura*	Control-vs-HIPVs-exposed	48	1.11	**< 0.01****
	S. frugiperda	Control-vs-HIPVs-exposed	48	0.23	**< 0.01****
Figure 1F	*S. litura*	Control-vs-HIPVs-exposed	48	0.01	0.07
	S. frugiperda	Control-vs-HIPVs-exposed	48	0.19	**< 0.01****
Figure 1G	*S. litura*	Control-vs-HIPVs-exposed	48	0.01	0.36
	S. frugiperda	Control-vs-HIPVs-exposed	48	0.30	**< 0.01****
Figure 1H	*S. litura*	Control-vs-HIPVs-exposed	48	0.002	0.62
	S. frugiperda	Control-vs-HIPVs-exposed	48	4.15	**< 0.01****

Asterisks indicate significant differences (*, P<0.05; **, P<0.01).

Table S3. Independent sample T-test and Two-way ANOVA result in Figure 2

Figure	Pest	Group	d.f.	*F* value	*P* value
Figure 2A	*S. litura*	Control-vs-HIPVs-exposed	48	3.25	0.84
	S. frugiperda	Control-vs-HIPVs-exposed	48	7.30	**< 0.01****
Figure 2B	*S. litura*	Control-vs-HIPVs-exposed	48	6.73	0.37
	S. frugiperda	Control-vs-HIPVs-exposed	48	4.56	**< 0.01****
Figure 2C	*S. frugiperda*	PBO(P)	1	0.51	**< 0.01****
		HIPV (H)	1	0.54	**< 0.01****
		P × H	1	4.73	**0.01****
Figure 2D	*S. frugiperda*	PBO(P)	1	1.02	**< 0.01****
		HIPV (H)	1	0.04	**< 0.01****
		P × H	1	2.39	**0.01****

Asterisks indicate significant differences (*, P<0.05; **, P<0.01).

Table S4. Independent sample T-test result in Figure 3

Figure	Pest	Group	d.f.	*F* value	*P* value
Figure 3A	*S. litura*	Control-vs-HIPVs-exposed	98	0.26	0.23
	S. frugiperda	Control-vs-HIPVs-exposed	98	0.12	0.16
Figure 3B	*S. litura*	Control-vs-HIPVs-exposed	98	7.87	0.31
	S. frugiperda	Control-vs-HIPVs-exposed	98	1.11	0.84
Figure 3C	*S. litura*	Control-vs-HIPVs-exposed	98	0.36	0.16
	S. frugiperda	Control-vs-HIPVs-exposed	98	1.157	0.31
Figure 3D	*S. litura*	Control-vs-HIPVs-exposed	98	0.82	0.84

	S. frugiperda	Control-vs-HIPVs-exposed	98	1.54	0.27

Asterisks indicate significant differences (*, P<0.05; **, P<0.01).

Table S5. Two-way ANOVA result in Figure 4

Figure	Pest	Group	d.f.	*F* value	*P* value
Figure 4A	*S. litura*	DIMBOA (D)	1	0.31	**< 0.01****
		HIPV (H)	1	2.51	**< 0.01****
		D × H	1	1.07	0.10
Figure 4B	*S. frugiperda*	PBO(P)	1	7.09	**< 0.01****
		HIPV (H)	1	2.44	**< 0.01****
		P × H	1	0.56	0.989
Figure 4C	*S. litura*	DIMBOA (D)	1	3.97	**< 0.01****
		HIPV (H)	1	4.78	**< 0.01****
		D × H	1	1.07	**< 0.01****
Figure 4D	*S. frugiperda*	PBO(P)	1	5.88	**< 0.01****
		HIPV (H)	1	3.65	**< 0.01****
		P × H	1	8.42	0.452
Figure 4E	*S. litura*	DIMBOA (D)	1	5.97	**0.01****
		HIPV (H)	1	3.27	**< 0.01****
		D × H	1	0.99	**0.01***
Figure 4F	*S. frugiperda*	PBO(P)	1	8.67	**< 0.01****
		HIPV (H)	1	1.34	**< 0.01****
		P × H	1	2.95	**0.08**
Figure 4G	*S. litura*	DIMBOA (D)	1	13.55	**0.01***
		HIPV (H)	1	1.92	0.124
		D × H	1	1.41	**0.03***
Figure 4H	*S. frugiperda*	PBO(P)	1	2.73	**< 0.01****
		HIPV (H)	1	5.59	**< 0.01****
		P × H	1	7.72	**0.02***
Figure 4I	*S. litura*	PBO(P)	1	0.26	**< 0.01****
		HIPV (H)	1	2.11	**< 0.01****
		P × H	1	6.85	**< 0.01****
Figure 4J	*S. frugiperda*	PBO(P)	1	5.66	**< 0.01****
		HIPV (H)	1	3.34	**< 0.01****
		P × H	1	6.10	**< 0.01****
Figure 4K	*S. litura*	PBO(P)	1	2.11	**< 0.01****
		HIPV (H)	1	3.43	**< 0.01****
		P × H	1	3.01	**0.01***
Figure 4L	*S. frugiperda*	PBO(P)	1	9.87	**< 0.01****
		HIPV (H)	1	6.52	**< 0.01****
		P × H	1	4.33	**< 0.01****

Asterisks indicate significant differences (*, P<0.05; **, P<0.01).

Table S6. Two-way ANOVA result in Figure 5

Figure	Pest	Group	d.f.	*F* value	*P* value
Figure 5A	*UGT33F28*-Midgut	DIMBOA (D)	1	1.88	**< 0.01****
		HIPV (H)	1	0.64	**< 0.01****
		D × H	1	0.31	**< 0.01****
Figure 5B	*UGT40L8*-Midgut	DIMBOA (D)	1	1.12	**< 0.01****
		HIPV (H)	1	0.47	**< 0.01****
		D × H	1	0.48	**< 0.01****
Figure 5C	*CYP4d8*-Midgut	DIMBOA (D)	1	2.91	**< 0.01****
		HIPV (H)	1	6.79	**< 0.01****
		D × H	1	15.75	**< 0.01****
Figure 5D	*CYP6B6*-Midgut	DIMBOA (D)	1	3.83	**< 0.01****
		HIPV (H)	1	0.54	**< 0.01****
		D × H	1	7.99	**< 0.01****
Figure 5E	*CYP4V2*-Midgut	DIMBOA (D)	1	0.43	0.206
		HIPV (H)	1	1.31	**< 0.01****
		D × H	1	8.39	0.346
Figure 5F	*UGT33F28*-Fatbody	DIMBOA (D)	1	7.66	**< 0.01****
		HIPV (H)	1	0.52	**0.111**
		D × H	1	3.09	**< 0.01****
Figure 5G	*UGT40L8*- Fatbody	DIMBOA (D)	1	1.31	**< 0.01****
		HIPV (H)	1	7.25	**0.02***
		D × H	1	0.23	**< 0.01****
Figure 5H	*CYP4d8*-Fatbody	DIMBOA (D)	1	4.86	**< 0.01****
		HIPV (H)	1	4.81	**< 0.01****
		D × H	1	2.83	**< 0.01****
Figure 5I	*CYP6B6*-Fatbody	DIMBOA (D)	1	0.47	**< 0.01****
		HIPV (H)	1	2.14	**< 0.01****
		D × H	1	2.23	**< 0.01****
Figure 5J	*CYP4V2*-Fatbody	DIMBOA (D)	1	0.82	**< 0.01****
		HIPV (H)	1	2.51	**< 0.01****
		D × H	1	7.63	0.24

Asterisks indicate significant differences (*, P<0.05; **, P<0.01).

Table S7. Independent sample T-test result in Figure 6

Figure	Pest	Group	d.f.	*F* value	*P* value
Figure 6A	*S. litura*	Control-vs-cis-HXO-exposed	38	13.92	**< 0.01****
	S. frugiperda	Control-vs-cis-HXO-exposed	38	0.038	**< 0.01****
Figure 6B	*S. litura*	Control-vs-cis-HXO-exposed	38	7.14	0.55
	S. frugiperda	Control-vs-cis-HXO-exposed	38	0.196	**< 0.01****

Asterisks indicate significant differences (*, P<0.05; **, P<0.01).

Table S8. Two-way ANOVA result in Figure 7

Figure	Pest	Group	d.f.	*F* value	*P* value
		DIMBOA (D)	1	1.45	< 0.01**
Figure 7A	*S. litura*	cis-3-HXO (H)	1	0.92	< 0.01**
		D × H	1	0.93	< 0.01**
		DIMBOA (D)	1	2.11	< 0.01**
Figure 7B	*S. frugiperda*	cis-3-HXO (H)	1	1.06	< 0.01**
		D × H	1	1.45	< 0.01**
		DIMBOA (D)	1	6.98	< 0.01**
Figure 7C	*S. litura*	cis-3-HXO (H)	1	12.57	< 0.01**
		D × H	1	3.82	0.55
		DIMBOA (D)	1	0.42	< 0.01**
Figure 7D	*S. frugiperda*	cis-3-HXO (H)	1	2.35	< 0.01**
		D × H	1	6.54	0.04
		DIMBOA (D)	1	9.19	< 0.01**
Figure 7E	*S. litura*	cis-3-HXO (H)	1	7.13	0.02*
		D × H	1	0.93	0.247
		DIMBOA (D)	1	5.25	< 0.01**
Figure 7F	*S. frugiperda*	cis-3-HXO (H)	1	8.38	0.04
		D × H	1	8.25	0.83
		DIMBOA (D)	1	6.17	< 0.01**
Figure 7G	*S. litura*	cis-3-HXO (H)	1	3.39	< 0.01**
		D × H	1	0.90	0.22
		DIMBOA (D)	1	4.41	< 0.01**
Figure 7H	*S. frugiperda*	cis-3-HXO (H)	1	0.45	< 0.01**
		D × H	1	0.29	0.02*
		DIMBOA (D)	1	5.26	< 0.01**
Figure 7I	*S. litura*	cis-3-HXO (H)	1	4.78	< 0.01**
		D × H	1	6.25	0.70
		DIMBOA (D)	1	0.15	< 0.01**
Figure 7J	*S. frugiperda*	cis-3-HXO (H)	1	0.22	< 0.01**
		D × H	1	3.32	0.76
		DIMBOA (D)	1	3.25	< 0.01**
Figure 7K	*S. litura*	cis-3-HXO (H)	1	1.21	0.00*
		D × H	1	1.85	0.01*
		DIMBOA (D)	1	13.85	< 0.01**
Figure 7L	*S. frugiperda*	cis-3-HXO (H)	1	12.76	0.03*
		D × H	1	1.45	0.49

Asterisks indicate significant differences (*, $P<0.05$; **, $P<0.01$).

Table S9. Two-way ANOVA result in Figure 8

Figure	Gene	Group	d.f.	*F* value	*P* value
		DIMBOA (D)	1	1.12	**< 0.01****
Figure 8A	*UGT33F28*-Midgut	cis-3-HXO (H)	1	5.52	**< 0.01****
		D × H	1	1.18	**< 0.01****
		DIMBOA (D)	1	0.59	**< 0.01****
Figure 8B	*UGT40L8*-Midgut	cis-3-HXO (H)	1	0.25	**< 0.01****
		D × H	1	0.62	**< 0.01****
		DIMBOA (D)	1	2.23	**< 0.01****
Figure 8C	*UGT33F28*-Fatbody	cis-3-HXO (H)	1	1.18	**< 0.01****
		D × H	1	3.57	**< 0.01****
		DIMBOA (D)	1	11.23	**< 0.01****
Figure 8D	*UGT40L8*- Fatbody	cis-3-HXO (H)	1	0.44	**< 0.01****
		D × H	1	2.15	**< 0.01****

Asterisks indicate significant differences (*, P<0.05; **, P<0.01).

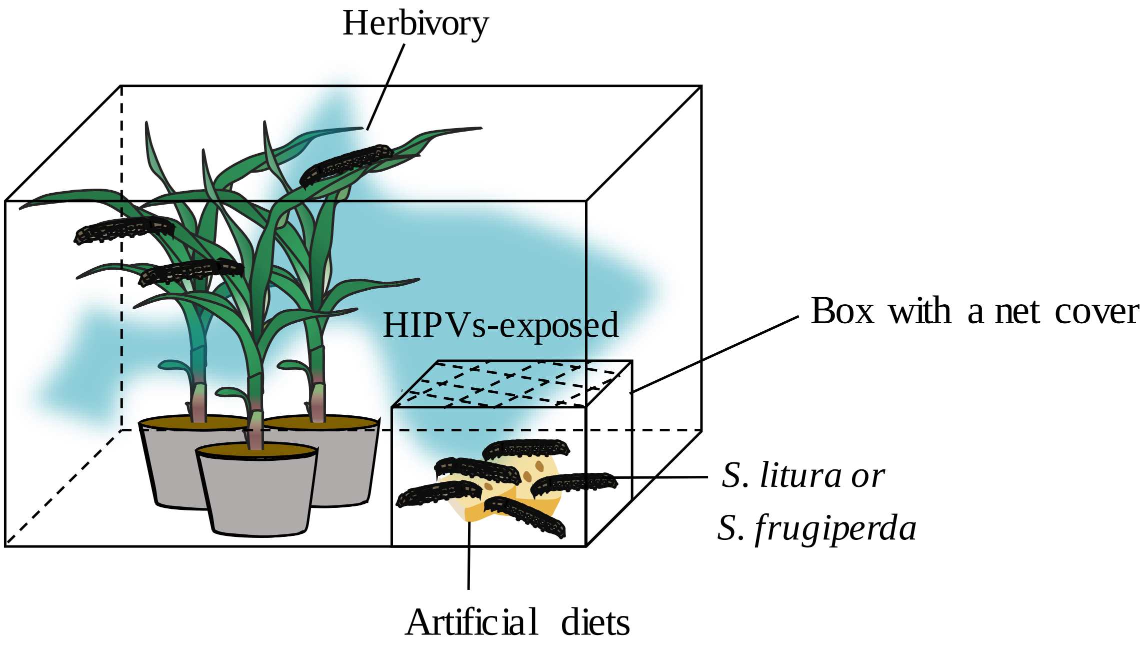

Figure S1. Schematic diagram showing the device of larval exposure of *Spodoptera frugiperda* and *S. litura* to herbivore-induced plant volatiles (HIPVs) from maize plants.

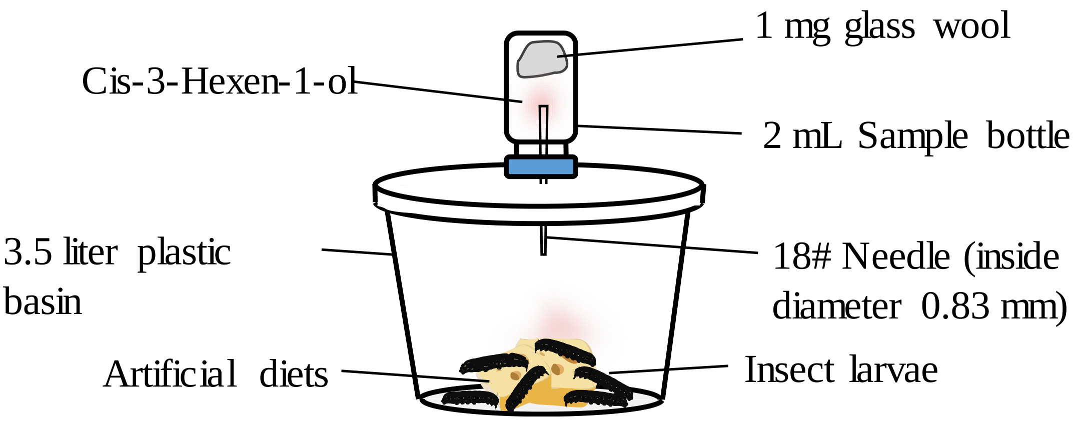

Figure S2. Schematic diagram showing the device of larval exposure of *Spodoptera frugiperda* and *S. litura* to volatile cis-3-hexen-1-ol.
